# Fertility-Sparing Methods in Adolescents Affected by Endometrial Cancer: A Comprehensive Review

**DOI:** 10.3390/jcm10051020

**Published:** 2021-03-02

**Authors:** Krzysztof Gałczyński, Piotr Olcha, Katarzyna Romanek-Piva, Maciej Jóźwik, Andrzej Semczuk

**Affiliations:** 1 Faculty of Medical Sciences and Health Sciences, Siedlce University of Natural Sciences and Humanities, Konarskiego Str. 2, 08-110 Siedlce, Poland; 2 Department of Gynecology, 1st Clinical Military Hospital in Lublin, Aleje Racławickie 23, 20-049 Lublin, Poland; piotrolcha@op.pl (P.O.); k.romanekpiva@gmail.com (K.R.-P.); 3 Department of Gynecology and Gynecological Endocrinology, Medical University of Lublin, Aleje Racławickie 23, 20-049 Lublin, Poland; 4 2nd Department of Gynecology, Medical University of Lublin, Jaczewskiego Str. 8, 20-954 Lublin, Poland; andrzej.semczuk@umlub.pl; 5 Department of Gynecology and Gynecologic Oncology, Medical University of Białystok, Skłodowskiej Str. 24A, 15-276 Białystok, Poland; jozwikmc@interia.pl

**Keywords:** adolescents, endometrial cancer, fertility, fertility preservation, young women

## Abstract

Although in developed countries endometrial cancer (EC) is the most common gynecological malignancy, its occurrence in adolescents is exceedingly rare. The increasing rate of obesity in children and adolescents is held responsible for the increasing prevalence of EC in younger cohorts of patients. The diagnosis of this malignancy can have devastating consequences for future fertility because standard treatment protocols for EC include hysterectomy. Here, we present the first detailed review of the world literature on EC in subjects aged 21 years or younger (*n* = 19). The mean age at diagnosis was 16.7 ± 0.6 years. One patient (5.3%) had a Type II (high-risk) disease. No communication retrieved from the search reported on patient death; however, two (10.5%) patients were lost to follow-up. There was also a high proportion (five subjects, or 26.3%) of cases with genetic background (Cowden syndrome and Turner syndrome), therefore genetic screening or a direct genetic study should be considered in very young patients with EC. The current fertility-sparing options, limited to Type I (low-risk) disease, are presented and discussed. Such information, obtained from studies on older women, translates well to adolescent girls and very young women. Careful anatomopathological monitoring at follow-up is essential for the safety of a conservative approach. Improved survival in very young EC patients makes the preservation of fertility a central survivorship issue, therefore both patients and caregivers should undergo counseling regarding available options. Moreover, our study suggests that genetic syndromes other than Lynch syndrome may be associated with EC more frequently than previously thought.

## 1. Introduction

The incidence and death rates reported for endometrial cancer (EC) demonstrate increases in 26 countries, with the greatest increments observed in North America and Western Europe [1]. According to the data published by the American Cancer Society^®^, the estimated numbers of new EC cases and EC-related deaths in the United States in 2020 will exceed 1,800,000 and 600,000, respectively. This translates into 4950 patients diagnosed with EC and 1660 patients who died from this malignancy daily [2]. Generally, EC is diagnosed in post- and perimenopausal women; however, recent reports state that approximately 7% of cases are diagnosed in young women aged 20 to 44 years [3]. It is expected that the incidence of EC in this age group will continue to be on the increase as a reflection of rising trends observed in the prevalence of EC risk factors such as obesity and diabetes mellitus [4,5]. It is difficult to provide precise estimations of the incidence of EC in the adolescent population because its occurrence in very young patients is exceedingly rare. However, the youngest patients reported in the literature were an 11-year-old [6] and a 13-year-old [7] girl.

To date, many EC risk factors have been identified and they also refer to the groups of adolescent and young patients. They include sedentary lifestyle, obesity, hyperinsulinemia and insulin resistance, type 2 diabetes mellitus, arterial hypertension, nulliparity, early menarche, anovulation and related conditions, polycystic ovary syndrome (PCOS), and hereditary cancer syndromes (such as Cowden syndrome and Lynch syndrome) [8,9,10,11]. Patients with Turner syndrome with spontaneous menarche also seem to carry a higher risk for EC [12,13]. Nonetheless, the most commonly identified risk factor in women with EC is obesity, which is a well-known source of excess estrogen. A high body mass index (BMI) directly correlates with increased risk of EC [3,14]. Nowadays, obesity is considered to be one of the crucial public health issues worldwide. The prevalence of obesity in children and adolescents has doubled in a single generation [8]. Diagnostic tools should be applied in young teenage girls complaining of abnormal genital bleeding, particularly in subjects with the aforementioned risk factors.

The typical precursor of EC is endometrial hyperplasia, an irregular proliferative process of the endometrial glands leading to an increased gland-to-stroma ratio. When cytologic atypia occurs in endometrial hyperplasia, then the risk for malignant transformation is high and the patient requires treatment. Women with EC or atypical endometrial hyperplasia (AEH) should undergo a total hysterectomy with bilateral salpingo-oophorectomy [15]. Furthermore, in the case of EC, when appropriate, the selective use of sentinel lymph node mapping or pelvic and para-aortic lymph node dissection should be performed depending on the risk factors and staging [15,16,17]. When EC occurs in adolescent or young women, in most cases the tumor represents a well-differentiated (Grade 1) endometrioid histological type, frequently focal and limited to the endometrium or superficial myometrium. As a tumor confined to the corpus uteri, it is thus allocated as stage I cancer in the Fédération Internationale de Gynécologie et d’Obstétrique, or FIGO, classification [15]. Reported oncologic outcomes in young women are excellent with 5-year disease-free survival rate of up to 99.2%. These outcomes strongly encourage fertility-sparing treatment in nulliparous women who desire pregnancy in the future [18]. Diminished reproductive capacity is a devastating consequence of life-sparing treatment for childhood malignancies; therefore, published recommendations underline the importance of early discussion and intervention for fertility-preservation strategies [16,19,20].

### 1.1. Fertility Issues in Adolescents and Young Adults with Cancer

Adolescence is a transitional stage of physical and psychological development that extends from puberty to legal adulthood. While the United Nations Convention on the Rights of the Child defines adolescence as the period between 10 and 19 years of age [21], there have been attempts to expand this timeframe to the period of 10–24 years [22]. The Medical Subject Headings (MeSH) vocabulary thesaurus provides the definition of an adolescent as a person 13 to 18 years of age [23]. In oncology, the term: ‘the adolescent and young adult (AYA) patient’ usually refers to individuals aged 15–39 years at the time of their initial diagnosis [24]. With this plethora of overlapping denominations, in this work we chose to specifically focus on AYA individuals diagnosed with EC aged 21 years and younger.

Regardless of their affliction, prognosis, and treatment, many AYAs diagnosed with cancer express a desire to have children in the future [25,26]. These patients rank having children among their top three life goals, along with health and education [27]. The improvement of treatment and survival of pediatric oncology patients has made the preservation of fertility a central survivorship issue [19]. The Declaration of Human Rights defines the right to a family as a basic human right [28]. Delivery of medical information before and during medical treatment is an essential duty of physicians. When a patient is incompetent or younger than the legal age, a legal guardian has the responsibility to decide on medical care [25,27,28]. Uncertainty occurs about how much adolescents should be involved in their disease management [25]. Counseling this group of patients regarding a fertility approach is challenging because of the delicate matter of the topic, among other things. First, discussing fertility issues with young patients may be discomforting for the physician. Second, parental protective buffering together with less developed executive functioning skills at a young age may disturb the process of giving informed consent. Studies of healthcare professionals show that the risk of infertility is not routinely discussed because of the lack of knowledge and training in this field, language and cultural barriers between the patient and the physician, the perception that the problem of fertility adds unnecessary stress to the already difficult situation, uncertainty about the success of fertility preservation methods or the patient’s ability to afford fertility preserving procedures, concerns about patients with an advanced disease and poor prognosis, and fear of delay of the initial treatment [28]. Young patients present a unique set of complexities while they undergo life-saving therapies, and they may be too young to fully understand the reproductive capacity-threatening consequences of oncologic therapies. Respecting the patient’s autonomy, which also applies to AYA patients, is one of the pillars of healthcare and obligates the physician to advocate for a fertility-sparing approach when possible [28]. Studies on adult survivors of cancer in adolescence identified the profound importance of addressing fertility concerns, which can affect family planning, personal well-being, and relationships. Young cancer survivors can experience depression and anxiety symptoms, grief, anger, lowered self-esteem, and an altered sense of identity related to reproductive problems [25,28,29]. Fertility information is pointed out by AYA survivors as one of the most common unmet needs. Previous studies showed that adolescents can cope with information about fertility options alongside a discussion of cancer. Women who did not receive fertility services in adolescence during cancer treatment reported great distress and regrets as adults. Limited knowledge of the impact of cancer treatment on fertility, together with a lack of information about fertility-sparing methods and the role of assisted reproductive technology (ART), may result in a situation where post-treatment infertility is a surprise and patients have unrealistic expectations about future childbearing. Empirical evidence revealed that children may be more capable of participating in their medical treatment than previously thought. When a clinician and the parents of a minor patient disagree about the content and amount of provided information regarding options for future fertility, multidisciplinary counseling should be considered [28,29].

### 1.2. Selection Criteria

According to the published data, when fertility-sparing options in the management of EC are considered, the patients should meet all of the five criteria mentioned below:

- Well-differentiated (Grade 1) endometrioid endometrial adenocarcinoma on dilatation and curettage confirmed by expert pathology review;
- Disease limited to the endometrium on magnetic resonance imaging (preferred) or transvaginal ultrasound;
- Absence of suspicious or metastatic disease on imaging;
- No contraindications to medical therapy or pregnancy;
- Counseling that fertility-sparing options are not a standard of care in the treatment of this cancer.

It is worth underlining that these guidelines are based on expert consensus opinion [1] or review papers [30], and not on prospective or randomized studies.

### 1.3. Existing Scarce Evidence

With a systematic review methodology at hand [31], we applied it to identify records of AYA patients with EC in the world literature. This search was conducted in line with the Preferred Reporting Items for Systematic Reviews and Meta-Analyses (PRISMA) group recommendations for systematic reviews [32]. Briefly, the literature was extensively explored electronically using online database searches from the earliest available records up to December 2020. The MEDLINE^®^ database was searched via PubMed^®^ search engine using the following MeSH-based terms, both alone and in combination: *women, female, adolescent, teenagers, youth, endometrial cancer, endometrial carcinoma*, *uterine cancer, reproduction, fertility*, and *fertility preservation*. This was supplemented by cross-referencing and manual searching (handsearching), since electronic databases can help identify only a portion of the available specialty data [33], and because early literature was frequently not covered electronically. Both non-English and English-language peer-reviewed articles were included. For inclusion, non-English articles had to have their abstracts in English. Duplicate hits were discarded. Unrelated studies were excluded through careful browsing of the title and abstract of each publication. All types of studies were selected, and each potentially relevant communication was obtained in full text and assessed for inclusion independently by at least three authors. Agreement for inclusion was reached by consensus.

All retrieved articles were in English. During the search it became apparent that scientific information regarding the occurrence of EC in AYAs, and specifically in adolescent patients, is not new as it was recognized in many early reports [6,34,35,36,37]. Later contributions described a 19-year-old woman with EC in endometrial polyp who later gave birth to a child who was 2 years old at her follow-up [38], or considered patients in an age range from 21 to 39 years [39]. Similarly, in this year’s prospective phase II trial on the medical treatment of AEH and early-stage EC, the youngest patient’s age was 19.1 years [40].

**Figure 1:**
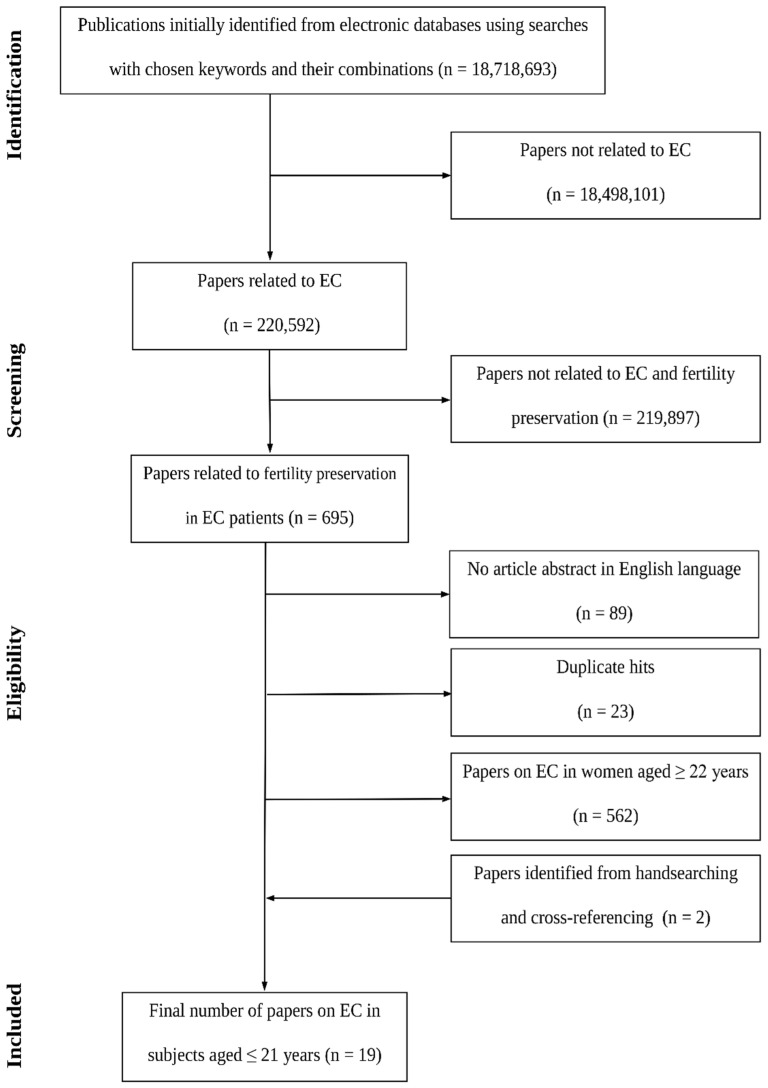
Study selection process for the systematic review of cases of endometrial cancer (EC) in subjects aged 21 years and younger.

Figure 1 presents the flowchart for the identification of relevant literature sources. Fifteen studies were identified as fulfilling the inclusion and exclusion criteria [7,11,12,13,41,42,43,44,45,46,47,48,49,50,51]. They presented 19 cases diagnosed with contemporary diagnostic criteria and described in sufficient or reasonably sufficient detail (Table 1). The mean reported age at diagnosis was 16.7 ± 0.6 years (mean ± SEM, range 13–21 years; *n* = 18). In general, except for a valuable series of patients studied by Farhi et al. [45], the literature consists of singular case studies. Abnormal genital bleeding, being the most frequent clinical complaint leading to the implementation of diagnostic steps, was present in 17 (89.5%) patients. One (5.3%) subject was taking oral contraceptives at the time of diagnosis [45], and one (5.3%) diagnostic exploration was done because of a polyp protruding through the external cervical os [42].

Notably, in 12 (63.2%) patients Grade 1 EC was diagnosed [11,13,41,44,45,46,49,51], in 5 (26.3%) patients Grade 2 (moderately differentiated) EC was diagnosed [7,42,43,48,50], and the diagnosis was not specified in 2 (10.5%) patients [12,47]. Endometrioid histology was ascribed to 16 (84.2%) cases, including 3 (15.8%) cases with concomitant AEH [7,45] and another 3 (15.8%) with concomitant areas of squamous differentiation [45,46]. Non-endometrioid histology (serous) was found in one (5.3%) patient [43]. Currently, prognosis for EC is being viewed on empirically established grounds whether a given case represents Type I or II EC, with Type I solely including endometrioid Grade 1 and 2 EC, and Type II comprising endometrioid Grade 3 and any other histotypes [15,52,53,54]. The prognosis for Type I EC is excellent, whereas it is much worse for Type II, depending on the cohorts studied. Therefore, it must be emphasized that at least one (5.3%) adolescent patient with EC, aged 17 years, had the Type II disease and it was a metastatic disease [43]. No communication retrieved from the search reported on patient death, however, two (10.5%) patients were lost to follow-up, one after radiation therapy and another who was known to have foci of cancer persisting after hormonal therapy (Table 1).

There were 3 (15.8%) cases with Cowden syndrome [41,44,50] and another 2 (10.5%) with Turner syndrome [12,13], yet the most frequent comorbidities were PCOS diagnosed in 7 (36.8%) patients [7,45,47,48,49] and increased body weight present in 6 (31.6%) [11,45,46,47,49,51]. A combination of PCOS and obesity was reported for 3 (15.8%) young women [45,47,49]. The most obese patient had a BMI of 63 [11].

The fertility-sparing approach was attempted in 11 (57.9%) patients, whereas others received surgical treatment at initiation of therapy or had subsequent surgery after failed medical treatment. Fertility-sparing modalities were mostly oral progestin given in varied, unstandardized daily doses (in 9 out of 11 patients, or 81.8%) and the application of a levonorgestrel (containing) intrauterine device (LNG-IUD) in 2 (18.2%) patients. The administration of 600 mg medroxyprogesterone acetate (MPA) per diem (p.d.) for 2 weeks only (followed by surgery) in Turner syndrome from the study by Ostor et al. [13] was not considered to be fertility-sparing. All in all, four cases of genetic syndromes and EC had surgical treatment, whereas in one case of Cowden syndrome a prolonged oral progestin administration was attempted [50].

Interestingly, four Grade 2 EC patients underwent fertility-sparing treatment. Brown et al. described the use of an LNG-IUD in an 18-year-old who was found free from disease at a 13-month follow-up [42]. Kim and colleagues presented a case of a 13-year-old girl suffering from menorrhagia and severe anemia. The patient received 160 mg megestrol acetate p.d. for 3 months, then 10 mg MPA p.d. for 5 months. A biopsy performed after 3 months of treatment revealed asynchronous endometrium with atypical glands. However, no residual tumor was found in subsequent biopsies. To support recovery, she was placed on an LNG-IUD [7]. Mitamura et al. reported on a 14-year-old girl with PCOS who received 400 mg of MPA daily for a month. Unfortunately, at follow-up, the endometrial biopsy revealed the presence of cancer cells; therefore, the patient underwent surgical treatment [48]. A patient from the Schmeler study underwent hysterectomy after the initial oral megestrol acetate (MA) therapy when the tumor’s grading was changed to Grade 2, a finding from the repeated endometrial biopsy that was confirmed in the final postoperative material [50].

The objective deficiencies of Table 1 testify to the need for a more careful detailed reporting of each case including the patient’s age at diagnosis, the clinical stage according to FIGO or other staging systems, the presence or absence of lymphovascular space invasion, sex hormone receptors’ status, and the outcome in the long-term follow-up.

There was a high proportion (26.3%) of cases with genetic background in our review. We underline this finding because, on the one hand, a lesser interest in fertility preservation in such cases may be expected. On the other hand, genetic screening or a direct genetic study should be given consideration in younger patients with EC. To date, various cases of Lynch syndrome, diagnosed on the basis of a germline mutation in *MLH1* and/or *MSH2* mismatch-repair genes, were associated with an earlier age at diagnosis and an increased lifetime risk of EC (by 40% to 60%) [54]. Interestingly, from a prospective multicenter study, the Lynch syndrome accounts for approximately 3% of all ECs, yet 9% of ECs in women under the age of 50 years [55].

## 2. Pharmacological Fertility-Sparing Treatment

Current pharmacological fertility-sparing modalities comprise hormonal therapies, mainly with progestins or progestin-releasing devices. Natural progesterone, aromatase inhibitors, oral contraceptives, selective estrogen receptor modulators (SERMs), and gonadotropin-releasing hormone (GnRH) agonists have also been used in EC, although the efficacy of progestin therapy is best known and described.

A mechanism of paramount importance in endometrial carcinogenesis is prolonged unopposed estrogen stimulation, which leads to both hyperplasia and cancer. Progesterone and progestins specifically act on the endometrium and protect cells against estrogen-driven growth and proliferation. This effect is exerted by several mechanisms which include downregulation of estrogen receptors (ERs), activation of enzymes involved in estrogen metabolism, regulation of the cell cycle by cyclin-dependent kinases, and enhancement of p27 expression, resulting in the inhibition of cyclin E-Cdk2 function and the suppression of the cell cycle [56,57,58]. As a result, progestins reverse endometrial hyperplasia and EC by stromal decidualization and the subsequent thinning of the endometrial lining [59].

### 2.1. Oral Progestins

The first successful use of progestins for fertility preservation was described in the 1990s [60]. Oral MPA and MA are the most commonly used for this purpose in EC patients. The reported response to the therapy is similar for the two drugs. However, there is a lack of detailed specific studies which compare the efficacy of MPA and MA in fertility-sparing therapy [56]. Some data show a significantly higher risk of recurrence after treatment with MA compared to therapy with MPA [61]. Previous studies most frequently used daily MPA doses, usually ranging from 200 to 800 mg (varied as much as 60 to 1800 mg) via different routes of administration, with most patients receiving doses equal to or greater than oral 400 mg p.d. When MA was applied, patients received it via different routes, from 40 to 480 mg p.d., yet most received less than 200 mg (again with a large dose variation from 10 to 480 mg). It is not clearly established whether low or high doses of progestin are more effective. Routinely, high empirical daily doses are prescribed. However, in studies comparing low- and high-dose MPA and MA for fertility-sparing treatment, no differences were found in the response and recurrence rates [56,60,62,63,64]. It needs to be underlined that high doses of progestins carry the risk of side effects and complications, together with an increased risk of non-compliance. Some authors reported a durable complete response in almost 90% of cases after the use of a low-dose regime (10–20 mg of MPA daily), yet their number of observations was low [61].

A profound impact of progestin therapy on the endometrial cells occurs as early as 10 weeks after initiation of treatment. Hence, the treatment duration before initial reassessment should last for at least 3 months, and at this timepoint the first results can be expected. When the disease is still present, but progression is not observed, the treatment should be extended to 12 months. If progression is found during the first assessment, surgical management should be performed. Published studies reported on progestin therapies of quite varied length: 3–36 months, with a median of approximately 6 months. It is widely accepted that the response should be evaluated by endometrial biopsy every 3 months. When a complete response is achieved, progestin therapy can be either maintained or discontinued, depending on the family plans of the patient. No specific guidelines exist regarding the duration of post-remission therapy. In obese and anovulatory patients who tend to be more resistant to therapy, a longer treatment should be considered [61,65]. Pregnancy attempts can be undertaken immediately. When cancer cells are still present 9 to 12 months after the initiation of the medical treatment, then radical treatment should be considered [56,66]. On average, complete response to the treatment occurs at circa 5.5 months of continuous use of the hormone. Reported complete response rates to such treatment range from 66% to 80%; however, subsequent recurrence occurs in 19% to 40% of cases. If so, this recurrence takes place after an average of 23 months. This observation underlines the fact that the primary goal of the conservative approach is to slow down the progression of the disease, which in turn allows for the patient to fulfill family plans before definitive surgical treatment [56,60,67,68,69]. When relapse occurs, a second cycle of progestin therapy can be implemented with a good response rate expected in up to 89% of the patients [16]. In a meta-analysis by Wei et al., a pooled complete response rate of 71% was reported in the group of patients with early-stage EC and complex AEH. Pooled pregnancy outcomes showed that 34% of women became pregnant, yet only 20% of them delivered live newborns [70].

A known side effect of progestin therapy is an increase in body weight. Several studies reported that pretreatment overweight or obesity was significantly associated with a poorer response to treatment and higher recurrence rate. Weight change (gain or loss) during progestin therapy was not associated with the recurrence rate in complete responders; however, BMI at the completion of the therapy was a significant predictor for recurrence [60,61]. Therefore, maintaining a normal BMI value is of utmost importance. Other adverse effects of oral progestin therapy include abdominal cramps, depression, dizziness, headaches, sleep disorders, venous thromboembolism and thrombophlebitis, and decreased libido [60,61].

### 2.2. Levonorgestrel Intrauterine Device

This device was designed in the 1980s in order to limit the above adverse effects of systemic progestin therapy. This treatment option also circumvents the patient non-compliance that accompanies oral medications [71]. All in all, the classical device delivers the amount of 52 mg of LNG directly to the target tissue (namely, the endometrium) over a period of 5 years, so the daily local release of the agent is miniscule.

In the initial months following insertion of the LNG-IUD, regions of the endometrium displaying secretory appearance are still obvious amongst the decidualized stroma, an observation that is consistent with progesterone-mediated transformation. With prolonged use, the morphological changes are much more uniformly distributed throughout the endometrium, producing an extensively decidualized and atrophic morphology [72]. The glandular epithelial cells become lower and thinner, and only solitary protrusions of these cells through the uniform basal lamina are observed [73]. Local LNG delivery results in diminished glandular expression and activity of prostaglandin dehydrogenase, a progesterone-responsive enzyme. High local concentrations of prostaglandins in the first 6 months after the insertion of the device are gradually reversed by decreased production and increased metabolism with continued progestin delivery [72]. This may be important provided that the expression of another intracellular enzyme cyclooxygenase-2 (or prostaglandin-endoperoxide synthase-2) markedly increases in cases of well-differentiated endometrial adenocarcinoma. On the one hand, cyclooxygenase-2 decreases apoptosis, and on the other hand, it participates in the synthesis of prostaglandin E_2_, which is known to regulate aromatase gene expression. Moreover, a number of studies described the induced immediate downregulation of the steroid receptors in the endometrium. After a month of exposure to the LNG-IUD, many ERs and progesterone receptors subtypes in both the glandular and stromal endometrial cells are already markedly reduced [74].

Despite the presence of the LNG-IUD in place, an endometrial biopsy can be performed without interfering with scheduled follow-ups. Complete response rates from 40% to 100% have been reported in premenopausal women with early-stage, well-differentiated EC [61]. Data from prospective studies indicate a greater regression of unbeneficial histology, lower relapse rates, and lower rates of subsequent hysterectomy when an LNG-IUD was used. Reports on the quality of life also demonstrate its superiority compared to oral progestins, with less sleep and mood disorders, fewer headaches, and less pronounced weight gain [16]. The pooled response rate for patients with an LNG-IUD was 76%, and the pooled recurrence rate was 9%. The pooled pregnancy rate was 18%, and 14% for delivering a live newborn. When a combined therapy of oral progestin plus LNG-IUD was used, then the pooled complete response rate was 87% in such patients, of whom 40% became pregnant and 35% delivered live progeny [70]. Therefore, this combined therapy requires further attention and broader studies.

A typical LNG-releasing intrauterine insert is T-shaped and has a 32 mm horizontal width and vertical length. Since 2013 in the USA, and since 2014 in many other countries, smaller similar devices with a 28 mm horizontal width and a 30 mm vertical length have become available. They were designed to match the internal dimensions of smaller uteruses, including those of very young women. Compact LNG-IUDs contain 13.5 mg of progestin, the duration of their use is 3 years, and their release rate of LNG is lower [75].

### 2.3. Anti-Estrogen Treatment

Currently used anti-estrogenic drugs are SERMs, selective estrogen receptor down-regulators (SERDs), and aromatase inhibitors (AIs). In the group of SERMs, raloxifene and arzoxifene (never marketed) block ERs, whereas tamoxifen exerts both blocking and stimulatory effects. The main SERD, fulvestrant, acts by downregulation of ERs. AIs, such as letrozole, decrease systemic exposure to estrogens by inhibition of the peripheral conversion of androgens to estrogens, mainly in fatty tissue. This process is a major source of excess estrogen in obese patients with EC. At present, limited evidence exists to support anti-estrogenic treatment(s) in fertility-sparing therapy [76,77]. Zhang et al. used a combination of a GnRH agonist with an AI in six obese patients with Grade 1 EC who wished to preserve fertility. Complete response was observed after 3 to 6 months in all patients. After a median follow-up of 4 years, none of the patients experienced recurrence. The pregnancy rate was 50%, weight gain was not observed during treatment, and the main side effects were menopause-like symptoms. The authors expressed the opinion that this combined therapy could be utilized as a primary treatment for obese EC patients or as the second resort after the failure of initial treatment with progestins alone [77].

### 2.4. Gonadotropin-Releasing Hormone Agonist

GnRH agonists decrease peripheral estrogen levels by the suppression of follicle-stimulating hormone and luteinizing hormone secretion. Since EC is frequently an estrogen-driven disease, this mechanism can be used when fertility sparing is considered. Moreover, 80% of ECs express GnRH and its receptors, which are specific low-affinity and high-capacity binding sites for both the hormone and its analogs. GnRH agonists reduce the proliferation rate of EC cells and are thought to interfere with the signal transduction of growth factor receptors and related oncogene products [78]. In 2003, Jadoul and Donnez were the first to report on the systematic combined use of partial hysteroscopic endometrial resection and GnRH agonist therapy as a conservative treatment for EC [78]. In 2018, Tock et al. retrospectively reviewed the experience of their center. GnRH agonist therapy was used for 3 months after surgery. Additionally, laparoscopy was performed in each case to exclude extrauterine disease. After a median follow-up of 40.7 months, 66.7% of patients had their uterus conserved, and 53.3% became pregnant among those who wished to become pregnant [79].

GnRH agonists can also be used together with an LNG-IUD. The complete response rate reported for such an option varies from 79% to 93%, with a subsequent pregnancy rate of 37%. When GnRH analogs are used, menopause-like symptoms can develop, the most serious side effect being bone loss. However, this risk is minimal if the therapy does not exceed the duration of 3–4 months [79].

## 3. Surgical Fertility-Sparing Treatment

### 3.1. Hysteroscopic Resection of Endometrium

Hysteroscopic surgical excision of the lesion with subsequent progestin therapy is another fertility-sparing option in patients with EC. One proposed approach is a three-step technique in which the tumor itself, the endometrium adjacent to the tumor, and then the underlying myometrium are removed one by one. Should the final pathology report confirm Grade 1 EC without myometrial invasion, then oral progestin therapy can be applied for 6 months. The reported efficacy for such an approach appears high. In a case series (*n* = 6) described by Mazzon et al., all patients experienced complete regression after 3 months of treatment and remained free from disease at the mean 50.5 months of follow-up. Four out of 6 patients had successful pregnancies without the need to resort to ART [80]. Laurelli et al. reported that, in 13 out of 14 similarly treated patients, no recurrence was observed 40 months after a complete response was achieved [81]. Yang et al. observed complete remission in 6 cases, without recurrence at the mean 32 months of follow-up, and one spontaneous pregnancy was achieved [82]. In the study by Shan et al., patients with EC or AEH received MA after complete hysteroscopic curettage. In total, 21 (80.8%) patients responded to the treatment. The median time to response was 12 weeks. Six patients had recurrence after a median follow-up of 32 months. Significantly, patients with a history of infertility or PCOS were more prone to experience recurrence. After complete response, 8 patients attempted to conceive, with the resultant 2 spontaneous conceptions and one normal delivery [83]. Giampaolino et al. described 14 patients with early EC who underwent hysteroscopic focal endometrial resection followed by an LNG-IUD insertion. In their study, complete response was observed in 78.6% of cases. This combined therapy showed similar response and live birth rates, but a considerably lower relapse rate, when compared with progestins alone [18]. The available body of evidence suggests that hysteroscopic tumor resection combined with subsequent progestin therapy is an effective and safe fertility-sparing modality for EC; however, larger studies are needed to confirm these results.

### 3.2. Ovarian Preservation

Ovarian preservation during hysterectomy provides a potential for future oocyte retrieval and surrogacy. According to the current state of knowledge, ovarian preservation has no effect on cancer-specific and overall survival. A decision regarding ovarian preservation should be individualized, since it carries a risk of missing occult synchronous ovarian cancer or metastases to the ovary. Even if the ovaries have an unsuspected macroscopical appearance, synchronous endometrial and ovarian cancer may exist [61]. The risk of synchronous ovarian cancer is higher in the cohort of young women with EC compared to older ones. For example, in one study, coexisting endometrial-ovarian malignancies were found in 25% of women aged 45 or younger, and in 2% of older patients [84]. It is estimated that such synchronous cancers occur in 5% of patients with EC and 10% of patients with ovarian cancer. Some authors suggest that these malignancies arise at one anatomic location and undergo restricted metastasis to the second site [71].

Important information was provided by a population-based analysis in the study of Gonthier et al. [85]. Young women with Grade 2 or 3 EC that was limited to the endometrium were compared in terms of survival following the surgery they had consented to: hysterectomy with bilateral salpingo-oopherectomy, or hysterectomy with ovarian preservation, or uterine preservation. Both the 5-year overall and cancer-related survival probabilities were much worse for uterine preservation than hysterectomy with or without oophorectomy. The authors concluded that ovarian preservation had no impact on survival probability in patients with Grade 2 or 3 EC confined to the endometrium [85].

## 4. Assisted Reproductive Technology

In cases when total hysterectomy with bilateral salpingo-oopherectomy is considered as the therapeutic option, ART can be applied before definitive treatment. The retrieval of oocytes for freezing, or directly for the in vitro fertilization of the retrieved eggs with subsequent freezing of the embryos, are the currently available options for women after menarche. Due to the introduction of vitrification techniques, oocytes survival and pregnancy rates have improved, and pregnancy rates and live births after the thawing and fertilizing of frozen eggs are currently reaching those obtained after embryo cryopreservation. For fertility preservation, random-start protocols are preferred because they shorten the time of oocyte retrieval to 2 weeks [86,87,88].

Ovarian tissue cryopreservation is another option for fertility preservation, and it can be applied both in women of reproductive age and prepubertal girls [86,89,90]. The procedure does not postpone oncological therapy and can be performed at any day of the cycle. Several methods for freezing gonadal tissues have been developed. Transplantation of frozen-thawed ovarian cortical tissue was demonstrated to provide recovery of ovarian functions and to restore spontaneous fertility. Successful hormonal stimulation with gonadotropins and in vitro fertilization have been reported in women who underwent retransplantation of ovarian tissue (so far, more than 200 live births worldwide) [27,86,87,91,92]. Initial reports on pregnancies resulting from transplanting ovarian tissue that was sampled and frozen in prepubertal years have also begun to appear [93].

## 5. Surveillance

The anatomopathological examination of endometrial tissue is required to assess the response to pharmacological therapy for EC. The most frequent approach is to start follow-up 3 months after the initiation of treatment, and then to perform subsequent endometrial sampling at 3-month intervals until complete response is achieved or progression of the disease occurs. Complete disease regression is not well defined: some physicians consider the presence of simple or complex hyperplasia without atypia as a complete regression, whereas some do not. Women who wish to delay pregnancy after remission should be placed on cyclic progestin therapy or an LNG-IUD because these decrease the risk of recurrence. After complete response, the patient should undergo follow-up surveillance every 3–6 months. Such controls include a pelvic examination, an endometrial biopsy, the determination of the levels of cancer antigen 125 in peripheral blood, and the evaluation of adnexa in imaging (by transvaginal ultrasound, computed tomography, or magnetic resonance) to exclude synchronous ovarian cancer. When recurrence occurs, it is confined to the uterus in the majority of cases; however, there have been reports of extrauterine spread even if complete response was achieved after initial treatment [61].

For many, the regression of EC with treatment and a subsequent pregnancy and safe delivery of the baby can be considered the final goal. However, even if rare, singular communications on the persistence of cancer cells in the endometrial curettage specimens after delivery at term [94] should be a cautionary tale for contemplating definitive surgery by the patient following childbearing. A report by Shiomi et al. reviewed 25 cases of EC diagnosed during or after pregnancy [95]. A straightforward recommendation of undergoing hysterectomy once the family has been completed was given as early as in 2003 [78].

## 6. Limitations

We acknowledge that there may be limitations of our literature search at both the outcome and review levels. Incomplete descriptions in many studies, particularly in early reports, may have hindered cases of EC in AYA patients. The titles and abstracts of such reports were insignificant and, as a consequence, our retrieval of identified research may be incomplete. Only the MEDLINE^®^ database was verified, and it is recognized that some articles may have been missed if not covered by this database.

Our extensive search documented only 19 relevant records, a limited number of observations. Therefore, all the results and conclusions of this work should be interpreted with caution. They are likely to be subject to change as further evidence accumulates.

## 7. Conclusions

Advances in the treatment of EC resulted in improved survival rates of adolescent and very young patients affected by this malignancy. In spite of this improvement, many therapeutic strategies can adversely and permanently impact the reproductive capacity of cancer survivors. The therapy should be tailored to the AYA patients’ characteristics and take into account its sequelae regarding sexual life, fertility, long-term side effects, follow-up regime, and adherence to the therapy. Regardless of the diagnosis, many adolescents diagnosed with cancer, together with their caregivers, express a desire to preserve fertility. Therefore, fertility-sparing strategies should be taken into consideration. The primary goal of this approach is to delay the progression of the disease which would allow for the patient to achieve pregnancy before definitive surgical treatment. Such information obtained from studies on older women translates well to adolescent girls and very young women. In this review of the world literature on 19 EC patients aged 21 years and younger, 1 (5.3%) patient had a Type II (high-risk) metastatic disease. There was also a high proportion (26.3%) of cases with genetic background, therefore genetic screening or a direct genetic study should be considered in very young patients with EC. Our study suggests that genetic syndromes other than Lynch syndrome, namely, Cowden syndrome and Turner syndrome, may be associated with EC more frequently than previously thought. Nonetheless, the available data demonstrates that fertility-sparing treatment of EC is safe and effective in well-selected (low-risk) groups of patients. When complete response in the medical treatment of EC is achieved, patients who wish to fulfill their desire for progeny should minimize the time to conception. When family plans are fulfilled, a hysterectomy should be contemplated.

## Figures and Tables

**Table 1:** A summary of the world literature on patients diagnosed with EC using contemporary diagnostic criteria and described in sufficient or reasonably sufficient detail, aged 21 years or younger (*n* = 19). Original descriptions of symptoms are cited.

Authors	Patient Age (Years), Comorbidities if Any	Symptom(s)	Anatomopathological Diagnosis	Treatment and Response
Baker et al. [41]	14, Cowden syndrome	Menorrhagia	Grade 1 EC	Surgical treatment upon patient’s request
Brown et al. [42]	18	Polyp protruding through the external cervical os	Grade 2 EC	Fertility-sparing approach, LNG-IUD
Cohn et al. [43]	17	Menorrhagia, secondary anemia	Grade 2 EC with serous metastasis	Surgical treatment
ElNaggar et al. [44]	15, Cowden syndrome	Excessive uterine bleeding	Grade 1 EC	Surgical treatment upon patient’s request
Farhi et al. [45]	21, obesity, PCOS	Irregular menses	Grade 1 EC, AEH present	Fertility-sparing approach with unspecified progestin
	15	Irregular menses	Grade 1 EC, AEH present	Fertility-sparing approach with unspecified progestin
	19, PCOS	Irregular menses, hirsutism	Grade 1 EC with areas of squamous differentiation,	Fertility-sparing approach with unspecified progestin, foci of cancer persisted after hormonal therapy, lost to follow-up
	15, PCOS	Irregular menses	Grade 1 EC,	Fertility-sparing approach: 400 mg MPA p.d. for 7 days, followed by weekly 400 mg intramuscular injections for 12 weeks, then 1 mg norethindrone acetate p.d. for 21 days of each month for 6 years, 2 successful term pregnancies, later vaginal hysterectomy
	21	On oral contraceptives	Grade 1 EC with areas of squamous differentiation	Radiation, lost to follow-up
Kim et al. [7]	13, PCOS	Irregular heavy menstrual bleedings, severe anemia	Grade 2 EC, AEH present; clinical stage IA	Fertility-sparing approach: 160 mg MA p.d. for 3 months, then 10 mg MPA for 5 months
Kocova et al. [12]	21, Turner syndrome	Prolonged heavy uterine bleeding	EC arising in a hyperplastic endometrial polyp	Fertility-sparing approach: MPA depot for 6 months, then radical treatment upon patient’s request
Koh et al. [46]	17, morbid obesity, arterial hypertension	A 2-day painless, heavy intermenstrual bleeding	Grade 1 EC with areas of squamous differentiation	Surgical treatment
Liu et al. [47]	15, obesity, PCOS	Persistent uterine bleeding	EC with myometrial invasion on MRI	Surgical treatment
Mitamura et al. [48]	14, PCOS	Menorrhagia	Grade 2 EC	Fertility-sparing approach: 400 mg MPA p.d. for a month, then surgical treatment
Ostor et al. [13]	19, Turner syndrome with mosaicism	Heavy menstruations at 2-month intervals, anemia, normal secondary sex characteristics	Grade 1 EC	600 mg MPA p.d. for 2 weeks only, then surgical treatment
Peterson [49]	16, morbid obesity since early childhood, PCOS	Prolonged periods of amenorrhea, extremely profuse menstrual bleedings	Grade 1 EC	Surgical treatment; follow-up at 21 years of age: free of recurrent or metastatic tumor
Rosen et al. [11]	17, morbid obesity as indicated by BMI = 63	Menorrhagia	Grade 1 EC	LNG-IUD; continues to be without evidence of disease
Schmeler et al. [50]	Adolescent (age not given), genetically confirmed familial *PTEN* mutation suggestive of Cowden syndrome in her case	Abnormal vaginal bleeding, pelvic pain	Grade 1 EC initially, then changed to Grade 2	Fertility-sparing approach: high-dose MA for 8 months, then robotic hysterectomy
Uda et al. [51]	14, overweight	Excessive uterine bleeding, severe anemia	Grade 1 EC	Fertility-sparing approach: 600 mg MPA p.d. for 26 weeks, complete response observed after 15 weeks

Abbreviations: AEH, atypical endometrial hyperplasia; EC, endometrial cancer; LNG-IUD, levonorgestrel (containing) intrauterine device; MA, megestrol acetate; MPA, medroxyprogesterone acetate; PCOS, polycystic ovary syndrome; p.d., per diem.

## Data Availability

All research data are openly available on proper request.
